# Clinical Characteristics of Geriatric Asthma in Guangxi of China: A Retrospective Comparative Descriptive Study

**DOI:** 10.1002/agm2.70083

**Published:** 2026-04-30

**Authors:** Mengxia Gu, Qixiang Sun, Yujia Huang, Yuetian Deng, Xiaohong Jiang

**Affiliations:** ^1^ Department of Geriatrics First People's Hospital of Nanning Nanning Guangxi China; ^2^ Department of Geriatric Respiratory Medicine The First Affiliated Hospital of Guangxi Medical University Nanning Guangxi China; ^3^ Department of Respiratory and Critical Care Medicine Baidong Campus of the Affiliated Hospital of Youjiang Medical College for Nationalities Baise Guangxi China; ^4^ Department of Endocrinology Medicine, Cadre Ward Guangxi Medical University Kaiyuan Langdong Hospital Nanning Guangxi China

**Keywords:** clinical characteristics, fixed airflow obstruction, geriatric asthma

## Abstract

**Objective:**

The research analyzed the clinical characteristics of geriatric and nongeriatric asthma cases, aiming to provide new insights into the diagnosis and management of geriatric asthma for clinicians.

**Methods:**

This retrospective study analyzed data from the First Affiliated Hospital of Guangxi Medical University database (2012–2022), including 130 geriatric (≥ 65 years) and 130 nongeriatric (18–64 years) asthma patients. Both cohorts were stratified into fixed (FAO) and reversible (RAO) airflow obstruction subgroups based on spirometry‐confirmed airflow limitation patterns. Demographic and clinical symptom data were extracted from electronic health records.

**Results:**

Geriatric asthmatics are mainly characterized by a greater prevalence in females (*n* = 78, 60%), increased cough (*n* = 123, 94.6%) and sputum (*n* = 116, 89.2%), poorer lung function (VC: 2.06 ± 0.65, FVC: 2.05 ± 0.64, FEV_1_:1.22 ± 0.49, PEF: 3.53 ± 1.59, all *p* < 0.05), elevated neutrophils ratios (66.7 ± 13.4, *p* < 0.05), and higher C‐reactive protein (CRP) levels (5.1 (11.1), *p* < 0.05). Geriatric asthmatics had longer average hospitalization days (9.9 ± 3.9, *p* < 0.05), higher sensitization rates to dust or irritant gases (*n* = 20, 15.4%, *p* < 0.05), and had more comorbidities compared to nongeriatric asthmatics. A history of asthma lasting > 20 years was identified as an independent risk factor for FAO in geriatric asthmatics (OR = 8.578, 95% CI = 1.610–45.741, *p* < 0.05).

**Conclusions:**

Geriatric asthmatics exhibit more comorbidities, poorer lung function, higher neutrophil ratios, and elevated CRP levels. They also experience longer hospital stays and are likely to be sensitized to dust or irritant gases. A longer duration of asthma significantly increases the risk of developing FAO in geriatric asthmatics.

## Graphical abstract

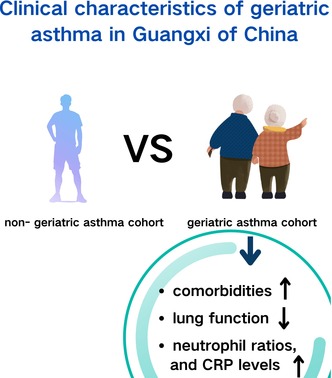

## 1 Introduction

Bronchial asthma is the most common chronic inflammatory respiratory disease worldwide, affecting 339 million people globally with a prevalence of 5%–10% [1]. According to 2022 data from the U.S. Centers for Disease Control and Prevention (CDC), the prevalence of asthma among adults aged 18 and older was 8.7%, while it was 8.0% in older adults aged 65 and above [2]. The 2022 World Population Prospects report predicted that by 2050, 1 in 6 (16%) people globally will be over the age of 65 [3]. With the accelerating aging population, the number of geriatric asthma patients is rising, imposing a heavy burden on the healthcare system and socioeconomic resources, particularly in low‐ and middle‐income countries [4, 5]. Geriatric asthma patients exhibit significantly higher mortality and hospitalization rates, healthcare costs, and reduced quality of life compared to nongeriatric asthma patients [6]. Geriatric asthma is defined as asthma occurring in individuals aged 65 years or older [7].

Compared to pediatric and adult asthma patients, geriatric asthma patients face markedly higher hospitalization and mortality rates, posing a serious health challenge for the older population [8]. Unlike young individuals, geriatric asthma patients often present with atypical clinical symptoms and signs. Additionally, age‐related declines in cognitive and sensory perception may lead to under‐recognition of asthma‐related discomfort by older patients [9]. Geriatric asthma is further complicated by frequent comorbidities and limitations in pulmonary function testing, all of which contribute to delayed or missed diagnosis [10].

Although variable and reversible airflow obstruction (RAO) is a defining feature of asthma, some patients develop irreversible airflow obstruction, with a Forced Expiratory Volume in the 1st second/Forced Vital Capacity ratio (FEV_1_/FVC) < 0.7 and an FEV_1_ improvement rate < 12% after using a bronchodilator, termed fixed airflow obstruction (FAO). Studies suggest that risk factors associated with FAO in geriatric asthma include male sex, advanced age, African American ethnicity, and prolonged asthma duration [11].

The study aims to summarize the clinical characteristics of geriatric asthma, analyze risk factors for FAO in this population, and evaluate the diagnostic value of peripheral blood parameters and pulmonary function tests in geriatric asthma, thereby providing new insights for its prevention and treatment. Compared to existing studies on geriatric asthma in China, the novelty of this study lies in its stratification of the asthma cohort into FAO and RAO subgroups for comparative analysis. Furthermore, when comparing the FAO subgroups between geriatric and nongeriatric asthma patients, a stratified analysis was conducted based on the presence or absence of COPD overlap. This approach preserved the integrity of the case data while mitigating potential confounding effects from COPD‐overlapping cases on the data analysis.

## 2 Materials and Methods

### 2.1 Patient Selection and Data Collection

This study is a comparative retrospective analysis utilizing data from the medical records database of the First Affiliated Hospital of Guangxi Medical University. We enrolled 130 asthma patients aged ≥ 65 years (geriatric asthma group) who were treated between January 2012 and August 2022, along with 130 contemporaneous asthma patients aged 18–64 years (nongeriatric asthma group) as controls. The diagnostic criteria for enrolled cases follow the 2023 Global Initiative for Asthma (GINA) guidelines and the Chinese Expert Consensus on the Diagnosis and Management of Asthma in Older Adults [7, 8, 9, 10, 11, 12]. The asthma cases enrolled in this study were divided into exacerbation and chronic persistent phases. For the concomitant diseases included in the cases, the diagnosis of Chronic Obstructive Pulmonary Disease (COPD) was based on the 2023 version of the GOLD Guidelines. Exclusion criteria were cases with key variables missing > 20%, asthma during pregnancy, severe cardiac dysfunction or cerebrovascular disease, and malignant tumors.

The participants were further stratified into fixed airflow obstruction (FAO) and reversible airflow obstruction (RAO) subgroups based on pulmonary function characteristics. Of the originally enrolled 130 matched pairs, complete pulmonary function data for subgroup classification were available in: 111 geriatric asthmatics (FAO: *n* = 82, RAO: *n* = 29) and 123 nongeriatric asthmatics (FAO: *n* = 71, RAO: *n* = 52). Among the cohort, 37 of 82 geriatric asthmatics presented with asthma‐COPD overlap (ACO), whereas only 3 of 71 nongeriatric asthmatic cases exhibited ACO. Given the established impact of comorbid COPD on fixed airflow obstruction (FAO) development in asthma, we performed stratified analyses when comparing FAO between geriatric and nongeriatric groups: one subset included ACO cases, and another excluded patients with COPD comorbidity. All subgroup analyses maintained the same statistical approaches as the primary analyses.

The Ethics Committee of the First Affiliated Hospital of Guangxi Medical University approved the data collection of this retrospective study and granted the exemption from informed consent (Approval Number: 2025‐E0047). Data analysis included demographic characteristics (gender, smoking history, body mass index (BMI), etc.), asthma‐related symptoms, comorbidities, therapeutic regimens during hospitalization, and laboratory parameters (blood biochemistry markers and pulmonary function measurements) for both geriatric and nongeriatric asthma cohorts.

### 2.2 Statistical Analysis

Data organization and graphical presentation were conducted using Microsoft Office Suite (Word, Excel, PowerPoint). Statistical analysis was performed with SPSS Statistics version 27.0. Normality was assessed for all continuous variables. For normally distributed data, two‐group comparisons used independent samples *t*‐tests, while multi‐group comparisons used one‐way ANOVA. Nonnormally distributed data were analyzed using nonparametric tests (Mann–Whitney *U* or Kruskal–Wallis tests as appropriate). Categorical variables were compared using chi‐square tests. Binary logistic regression was applied to identify risk factors associated with fixed airflow obstruction (FAO) in the geriatric asthma cohort. A two‐tailed *p*‐value < 0.05 defined statistical significance.

## 3 Results

### 3.1 Baseline Characteristics

The geriatric asthma cohort demonstrated female predominance (60%, *n* = 78), with 26.16% being current/former smokers. The mean age at asthma diagnosis was 25.1 years higher in the geriatric group (58.0 ± 20.2 years vs. 32.9 ± 14.6 years in the nongeriatric group; *p* < 0.001), suggesting that late‐onset asthma may be a predominant phenotype in geriatric patients. Obesity (BMI ≥ 28 kg/m^2^) was observed in 8.46% of geriatric patients. Cough and sputum production constituted primary symptoms in older adults, whereas wheezing (*p* < 0.001) and chest tightness (*p* = 0.027) were less prevalent compared to younger counterparts. A significantly higher proportion of geriatric asthma patients had an asthma history exceeding 20 years compared to nongeriatric patients (*p* = 0.003). Key intergroup comparisons revealed significantly prolonged hospitalization duration (*p* = 0.001) and higher sensitivity to dust/irritant gas allergens (*p* = 0.004) in the geriatric group. Comorbidity burden was substantially greater in geriatric patients, with significantly higher prevalence rates of chronic obstructive pulmonary disease (COPD) (*p* = 0.000), chronic bronchitis (*p* = 0.000), hypertension (*p* = 0.000), coronary heart disease (*p* = 0.019), diabetes mellitus (*p* = 0.001), and dyslipidemia (*p* = 0.042). No significant intergroup differences were observed in gender distribution, smoking history, obesity rates, food/drug allergies, family history of asthma, or occupational exposures. Demographic characteristics, asthma symptoms, comorbidities, and laboratory/pulmonary function parameters of 130 geriatric and 130 nongeriatric asthma patients are presented in Table 1a.

**TABLE 1a:** Characteristics of geriatric asthma cohort and nongeriatric asthma cohort.

Variables	Geriatric asthma (*n* = 130)	Nongeriatric asthma (*n* = 130)	*p*
Gender, *n* (%)			
Male	52 (40.0)	60 (46.2)	0.316
Female	78 (60.0)	70 (53.8)	
Age, mean ± SD	72.0 ± 6.1	40.4 ± 11.1	—
Age at diagnosis	58.0 ± 20.2	32.9 ± 14.6	< 0.001
Ex‐smokes or smokers, *n* (%)	34 (26.2)	34 (26.2)	—
Obesity (BMI ≥ 28 kg/m^2^), *n* (%)	11 (8.5)	9 (6.9)	0.642
Hospitalization duration, mean ± SD	9.9 ± 3.9	8.3 ± 3.6	0.001
History of food/drug allergy, *n* (%)	38 (29.2)	45 (34.6)	0.352
Dust/irritant gas sensitization, *n* (%)	20 (15.4)	6 (4.6)	0.004
History of occupational exposure, *n* (%)	3 (2.3)	8 (6.2)	0.216
Family history, *n* (%)	24 (18.5)	22 (16.9)	0.745
Asthma history > 20 years	38 (29.2)	18 (13.8)	0.003
Asthma status at inclusion			
Exacerbation	109 (83.8)	119 (91.5)	0.060
Chronic persistent phase	21 (16.2)	11 (8.5)	
Symptom, *n* (%)			
Cough	123 (94.6)	121 (93.1)	0.606
Expectoration	116 (89.2)	111 (85.4)	0.352
Respite	83 (63.9)	108 (83.1)	< 0.001
Chest tightness	37 (28.5)	54 (41.5)	0.027
Comorbidities, *n* (%)			
Allergic rhinitis	28 (21.5)	40 (30.8)	0.090
Sinusitis	11 (8.5)	31 (23.9)	0.001
Chronic pharyngitis	5 (3.9)	7 (5.4)	0.554
COPD	40 (30.8)	4 (3.1)	0.000
History of eczema/urticaria/topic dermatitis	12 (9.2)	15 (11.5)	0.542
Chronic bronchitis	22 (16.9)	2 (1.5)	0.000
Bronchiectasis	13 (10.0)	15 (11.5)	0.689
Pneumonia	74 (56.9)	75 (57.7)	0.900
Tuberculosis	13 (10.0)	6 (4.6)	0.095
Gastroesophageal reflux	5 (3.9)	1 (0.8)	0.213
Depression	3 (2.3)	0 (0.0)	0.247
Anxiety	1 (0.8)	2 (1.5)	1.000
Sleep disorders	7 (5.4)	1 (0.8)	0.066
OSAHS	3 (2.3)	0 (0.0)	0.247
Hypertension	67 (51.5)	12 (9.2)	0.000
Coronary heart disease	9 (6.9)	1 (0.8)	0.019
Dyslipidemia	38 (29.2)	24 (18.5)	0.042
Diabetes	22 (16.9)	6 (4.6)	0.001
Hematological profile, mean ± SD			
White blood cell count (^10^9^/L)	8.7 ± 3.8	9.4 ± 2.9	0.100
Red blood cell count (^10^12/^L)	4.4 ± 0.7	4.9 ± 0.6	0.000
Platelet count (^10^9^/L)	245.3 ± 72.7	273.1 ± 64.2	0.001
Lymphocyte count (^10^9^/L)	1.7 ± 0.7	2.2 ± 0.9	0.000
Neutrophil count (^10^9^/L)	6.0 ± 3.5	5.8 ± 2.7	0.525
Neutrophil ratio (%)	66.7 ± 13.4	59.9 ± 13.9	0.000
Eosinophil count (^10^9^/L), Median (IQR)	0.2 (0.3)	0.5 (0.8)	0.000
C‐reactive protein (mg/L), median (IQR)	5.1 (11.1)	3.0 (6.9)	0.011
Therapeutic patterns, *n* (%)			
Treated with ICS‐LABA	127 (97.7)	106 (81.5)	< 0.001
Treated with systemic corticosteroids	42 (32.3)	52 (40.0)	0.197

*Note:* According to the 2024 Chinese Guidelines for the Diagnosis and Treatment of Obesity, a BMI ≥ 28 kg/m^2^ can be diagnosed as obesity [13]. Hospital duration refers to the number of days a patient was last hospitalized for asthma, recorded in the hospital's case database. Occupational exposure history refers to long‐term exposure to dust or irritating gases in the working environment.

Abbreviations: BMI, body mass index; COPD, chronic obstructive pulmonary disease; ICS‐LABA, Inhaled corticosteroids and Long‐acting Betaagonists; OSAHS, Obstructive Sleep Apnea‐Hypopnea syndrome.

### 3.2 Hematological Profile

Geriatric patients exhibited elevated neutrophil percentages and CRP levels versus controls (all *p* < 0.05), alongside reduced lymphocyte counts, eosinophil counts, erythrocyte counts, and platelet counts. White blood cell and neutrophil counts showed no statistical divergence.

### 3.3 Pulmonary Function

Comparative analysis of pulmonary function parameters between the geriatric cohort (*n* = 111) and nongeriatric cohort (*n* = 123) revealed significant differences in the original data analysis (Table 1b). The geriatric group showed significantly lower values in vital capacity (VC), forced vital capacity (FVC), Forced Expiratory Volume in the 1st second (FEV_1_), peak expiratory flow (PEF), PEF% predicted, post‐bronchodilator FEV_1_/FVC ratio, and diffusing capacity of the lung for carbon monoxide percent predicted (DLCO%pred), while demonstrating significantly higher residual volume to total lung capacity ratio (RV/TLC) (all *p* < 0.05). No significant between‐group differences were observed in FEV_1_% predicted and pre‐bronchodilator FEV_1_/FVC ratio.

**TABLE 1b:** Pulmonary function of geriatric asthma cohort and nongeriatric asthma cohort.

Variables	Geriatric asthma (*n* = 111)	Nongeriatric asthma (*n* = 123)	*p*
VC (L)	2.06 ± 0.65	3.02 ± 0.88	< 0.001
FVC (L)	2.05 ± 0.64	3.00 ± 0.93	< 0.001
FEV_1_ (L)	1.22 ± 0.49	1.96 ± 0.80	< 0.001
FEV1% prediction	65.81 ± 22.94	65.40 ± 23.84	0.895
PEF (L/S)	3.53 ± 1.59	4.96 ± 2.12	< 0.001
PEF% prediction	60.71 ± 24.59	67.38 ± 25.95	0.045
Pre‐bronchodiator FEV_1_/FVC (%)	59.70 ± 14.42	63.29 ± 15.20	0.066
post‐bronchodilator FEV_1_/FVC (%)	61.85 ± 12.61	66.04 ± 13.83	0.017
DLCO%pred	73.79 ± 26.55	88.24 ± 26.56	< 0.001
RV/TLC (%)	49.93 ± 13.28	36.3 ± 9.94	< 0.001

*Note:* This table is based on the analysis of cases with complete pulmonary function data.

Abbreviations: DLCO%pred, diffusing capacity of the lung for carbon monoxide percent predicted; FEV_1_, forced expiratory volume in the 1st second; FVC, forced vital capacity; FVC, forced vital capacity; PEF, peak expiratory flow; RV, residual volume; TLC, total lung capacity; VC, vital capacity.

To validate the robustness of these findings, we performed multiple imputation analysis for missing data (14.6% in the geriatric cohort, 5.4% in the nongeriatric cohort). The imputed data (*n* = 130 per group) showed minimal differences from original results (< 3% mean difference), with all significant findings remaining consistent (unchanged *p*‐value direction and magnitude). Effect size analysis demonstrated stable results for key parameters (Δ *d* < 10%). Quality indicators of imputation (FMI < 0.20, RE > 0.95) further supported the reliability of the original data. Detailed results of multiple imputation analysis are provided in Tables S1 and S2.

### 3.4 Therapeutic Patterns

ICS‐LABA (inhaled corticosteroid‐long‐acting β₂ agonist) combination therapy was administered to 97.7% (*n* = 127) of geriatric patients. No significant therapeutic disparities were observed between groups regarding ICS‐LABA utilization or systemic corticosteroid requirements.

### 3.5 Subgroup Analysis

**FIGURE 1:**
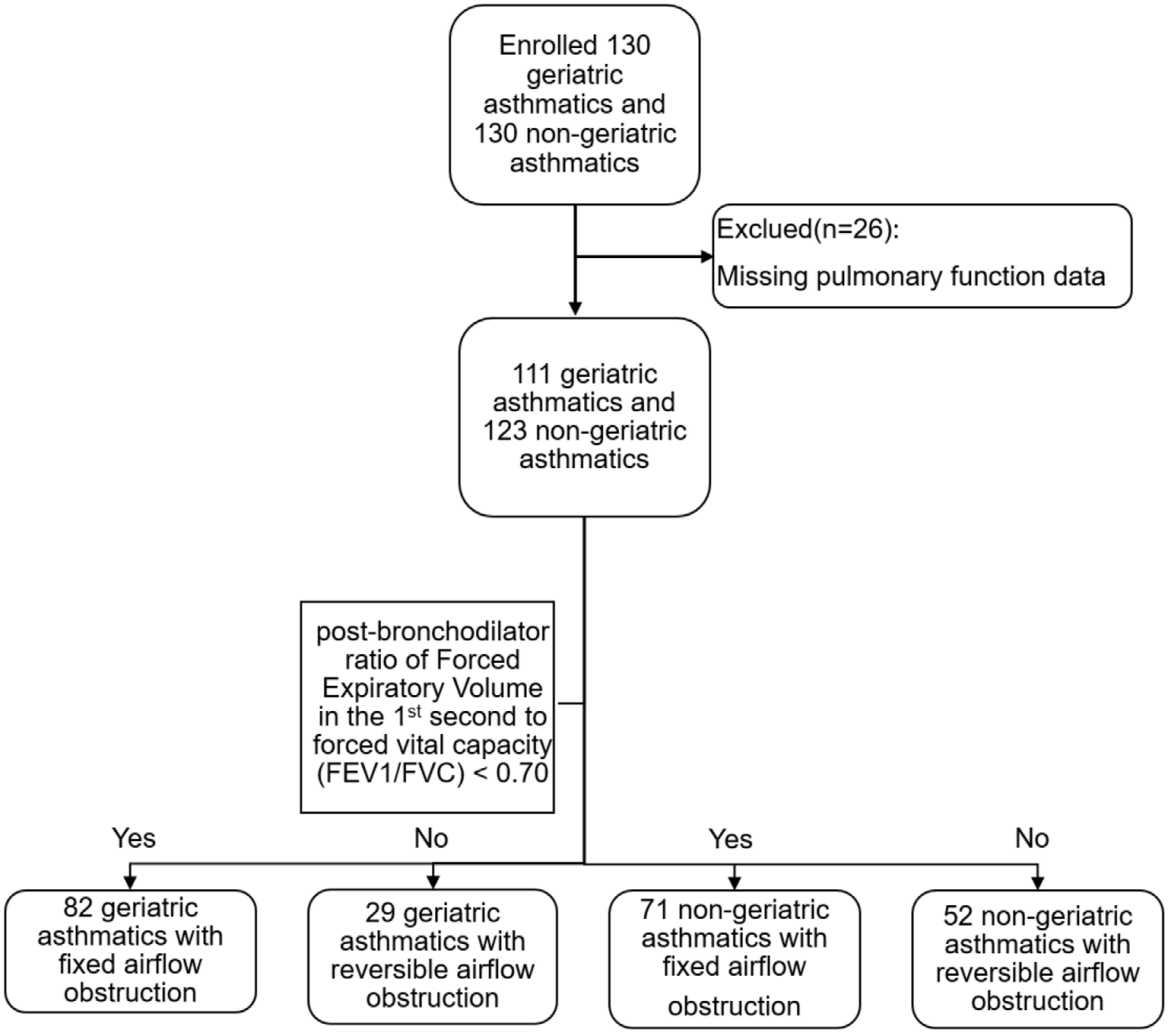
Flowchart of the screening process for the FAO group and RAO group. FAO, Fixed airflow obstruction; RAO, Reversible airflow obstruction.

We applied the most widely accepted diagnostic criterion for FAO: post‐bronchodilator ratio of Forced Expiratory Volume in the 1st second to forced vital capacity (FEV_1_/FVC) < 0.70. The geriatric asthma cohort was subdivided into FAO (*n* = 82) and RAO (*n* = 52) subgroups based on spirometric confirmation of airflow limitation patterns. Figure 1 illustrates the screening algorithm for stratifying patients into fixed airflow obstruction (FAO) and reversible airflow obstruction (RAO) subgroups. Table 2 presents the baseline characteristics of the FAO and RAO groups in geriatric asthmatics. Table 3a presents the characteristics of FAO groups in geriatric asthmatics and nongeriatric asthmatics (cases containing asthma‐COPD overlap). We found that the geriatric asthma FAO group had a longer asthma course (*p* < 0.001) and were more likely to develop dust/irritating gas allergies (*p* = 0.003) than the nongeriatric asthma FAO group. Pulmonary function assessments showed significantly reduced DLCO%pred (*p* < 0.05) in the FAO groups in geriatric asthmatics. Conversely, the residual volume to total lung capacity ratio (RV/TLC) was significantly elevated (*p* = 0.002), indicating air trapping severity (Table 3b).

**TABLE 2:** Characteristics of Fixed airflow obstruction (FAO) and Reversible airflow obstruction (RAO) groups in geriatric asthmatics.

Variables	FAO (*n* = 82)	RAO (*n* = 29)	*p*
Age at baseline, mean ± SD, years	72.5 ± 6.1	71.6 ± 6.5	0.497
Male, *n* (%)	37 (45.1)	7 (24.1)	0.047
BMI, mean ± SD kg/m^2^	22.9 ± 3.3	24.7 ± 2.7	0.009
Ex‐smokes or smokers, *n* (%)	25 (30.5)	3 (10.3)	0.045
History of food or drug allergies, *n* (%)	29 (35.4)	5 (17.2)	0.069
Dust/irritant gas sensitization, *n* (%)	12 (14.6)	3 (10.3)	0.755
Family history, *n* (%)	15 (18.3)	2 (6.9)	0.230
Asthma history > 20 years	36 (43.9)	2 (6.9)	0.000
Comorbidities, *n* (%)			
Rhinitis	22 (26.8)	7 (24.1)	0.513
Pneumonia	47 (57.3)	16 (55.2)	0.841
COPD	37 (45.1)	0 (0)	0.000
Hypertension	38 (46.3)	20 (69.0)	0.047
Coronary heart disease	3 (3.7)	4 (13.8)	0.075
Diabetes	14 (17.1)	4 (13.8)	0.777
Lung function			
FEV_1_% prediction	57.93 ± 19.58	88.12 ± 16.15	0.000
FEV_1_/FVC (%) before using bronchodilators	53.52 ± 10.90	77.20 ± 6.67	0.000
FEV_1_/FVC (%) after using bronchodilators	56.06 ± 8.90	78.23 ± 4.35	0.000
DLCO%pred	70.96 ± 27.67	81.78 ± 21.58	0.036
RV/TLC (%)	52.23 ± 12.80	43.40 ± 12.61	0.002

Abbreviations: BMI, body mass index; COPD, chronic obstructive pulmonary disease; DLCO, Diffusion Lung CO; FAO, fixed airflow obstruction; FEV_1_, forced expiratory volume in 1 s; FVC, forced vital capacity; RAO, reversible airflow obstruction; RV, residual volume; TLC, total lung capacity.

**TABLE 3a:** Characteristics of FAO groups in geriatric asthmatics and nongeriatric asthmatics (Cases containing asthma‐COPD overlap).

Variables	FAO geriatric asthma	FAO nongeriatric asthma	*p*
Cases, *n* (%)	82 (73.9)	71 (57.7)	0.010
Male, *n* (%)	37 (45.1)	36 (50.7)	0.491
Ex‐smokers or smokers, *n* (%)	25 (30.5)	20 (28.2)	0.754
History of food/drug allergies, *n* (%)	29 (35.4)	21 (29.6)	0.446
Dust/irritant gas sensitization, *n* (%)	12 (14.6)	1 (1.4)	0.003
Family history, *n* (%)	15 (18.3)	15 (21.1)	0.660
Asthma history > 20 years	36 (43.9)	11 (16.2)	< 0.001
*Lung function*			
FEV_1_% prediction	57.93 ± 19.58	52.59 ± 19.58	0.095
FEV_1_/FVC (%) before using bronchodilators	53.52 ± 10.90	53.63 ± 10.57	0.946
FEV_1_/FVC (%) after using bronchodilators	56.06 ± 8.90	56.63 ± 9.74	0.705
DLCO% prediction	70.96 ± 27.67	80.53 ± 24.77	0.027
RV/TLC (%)	52.23 ± 12.80	39.41 ± 10.61	< 0.001

Abbreviations: DLCO%pred, diffusing capacity of the lung for carbon monoxide percent predicted; FAO, fixed airflow obstruction; FEV_1_, forced expiratory volume in 1 s; FVC, forced vital capacity; RV, residual volume; TLC, Total Lung Capacity.

**TABLE 3b:** Characteristics of FAO groups in geriatric asthmatics and nongeriatric asthmatics (Exclude cases of asthma‐COPD overlap).

Variables	FAO geriatric asthma	FAO nongeriatric asthma	*p*
Cases, *n* (%)	45 (40.5)	68 (55.3)	0.030
Male, *n* (%)	14 (31.1)	33 (48.5)	0.066
Ex‐smokers or smokers, *n* (%)	9 (20.0)	18 (26.5)	0.430
History of food/drug allergies, *n* (%)	18 (40.0)	19 (27.9)	0.181
Dust/irritant gas sensitization, *n* (%)	4 (8.9)	1 (1.5)	0.081
Family history, *n* (%)	8 (17.8)	14 (20.6)	0.712
Asthma history > 20 years	17 (37.8)	11 (16.2)	0.009
*Lung function*			
FEV_1_% prediction	63.41 ± 21.17	53.55 ± 19.18	0.007
FEV_1_/FVC (%) before using bronchodilators	56.43 ± 10.27	53.70 ± 10.64	0.178
FEV_1_/FVC (%) after using bronchodilators	58.43 ± 8.23	56.75 ± 9.81	0.343
DLCO% prediction	75.92 ± 28.19	81.46 ± 24.33	0.269
RV/TLC (%)	50.46 ± 12.51	38.99 ± 10.58	< 0.001

When excluding ACO cases, geriatric FAO asthma is characterized by predominant air trapping (RV/TLC) and longer disease duration, rather than impaired diffusion capacity or higher sensitization rates. Air trapping (RV/TLC) is a persistent pathological feature in geriatric FAO asthma, independent of ACO comorbidity.

### 3.6 Risk Factors for Fixed Airflow Obstruction in Geriatric Asthma

Binary logistic regression analysis identified significant predictors of fixed airflow obstruction (FAO) development in geriatric asthma patients (Table 4). Unadjusted models revealed that **a**sthma duration > 20 years (OR = 10.565, 95% CI: 2.355–47.401, *p* = 0.002), COPD history (OR = ∞, 95% CI: 11.900–∞, *p* < 0.001), and smoking history (OR = 3.801, 95% CI: 1.052–13.730, *p* = 0.042) were key risk factors. After adjusting for potential confounders (including gender, age, and smoking history), prolonged asthma duration (> 20 years) remained a significant independent predictor of FAO (adjusted OR = 8.578, 95% CI: 1.610–45.741, *p* = 0.012), indicating a strong association between disease chronicity and irreversible airflow limitation. Although a history of COPD was significantly associated with FAO risk in univariate analysis, complete data separation precluded reliable estimation of its independent effect in the multivariable model. Neither male sex nor advanced age demonstrated a significant independent association with FAO risk in this cohort.

**TABLE 4:** Regression analysis of risk factors for the development of fixed airflow obstruction in geriatric asthmatics.

Variables	Model before correction		Model after correction	
	OR (95% CI)	*p*	OR (95% CI)	*p*
Male	2.584 (0.994–6.717)	0.051	1.812 (0.561–5.887)	0.321
Age	1.025 (0.955–1.100)	0.493	1.001 (0.923–1.086)	0.966
Asthma history > 20 years	10.565 (2.355–47.401)	0.002	8.578 (1.610–45.741)	0.012
History of smoking	3.801 (1.052–13.730)	0.042	2.702 (0.577–12.693)	0.212
COPD	∞ (11.900–∞)	< 0.001	—	—

*Note:* Since there were no patients with COPD in the geriatric asthma RAO group, complete data separation occurred, and the OR value was infinite (∞). Before adjusting for confounding factors, the *p*‐value and the lower limit of the 95% CI could be calculated through exact logistic regression analysis. However, after adjusting for confounding factors, OR and *p*‐values could not be calculated because the data were completely separated.

## 4 Discussion

Bronchial asthma, a heterogeneous disease characterized by chronic airway inflammation, affects 5%–10% of the global population [7]. Historically perceived as a childhood/adolescent disorder, epidemiological investigations reveal an increasing asthma prevalence among adults ≥ 65 years. Analysis of 61,815 asthma‐related deaths in the United States (1999–2015) demonstrated a mortality rate of 1.5 per 100,000 population, with significantly higher mortality in the > 65 age stratum compared to younger groups (1–14, 15–44, 45–64 years) [14]. A seminal Chinese cross‐sectional study (2010–2012, *n* = 164,215) further corroborated elevated mortality risks in geriatric asthma patients, associated with poorer Asthma Control Test (ACT) scores and multifaceted clinical challenges including atypical presentations, polypharmacy, inhaler technique deficiencies, and diagnostic complexities [15].

Guangxi, China, has entered the stage of a super‐aged society, characterized by a high proportion of elderly residents. This demographic situation underscores a significant increase in both the absolute number of geriatric asthma patients and their associated healthcare demands, which justifies our selection of Guangxi as the study site. Our retrospective analysis of 130 geriatric asthma inpatients (2012–2022) at the First Affiliated Hospital of Guangxi Medical University identified distinct clinical features: female predominance, cough/sputum‐dominant symptomatology, impaired pulmonary function (reduced VC, FVC, FEV_1_, PEF), elevated neutrophilic inflammation (neutrophil percentage, CRP), prolonged hospitalization, heightened sensitization to particulate/irritant allergens, and increased comorbidities (COPD, chronic bronchitis, hypertension, CAD, diabetes, and dyslipidemia).

Asthmatic airway inflammation and hyperresponsiveness are mediated through epithelial cell depletion, perpetuating chronic inflammatory states in geriatric airways [16, 17]. A 2018 Argentinian retrospective study demonstrated 96% sensitization prevalence in geriatric asthmatics (> 60 years), with dust mites (70%) and pollen (32%) as predominant allergens—aligning with our findings of environmental hypersensitivity [18].

Extended hospitalization durations reflect heightened disease burden in geriatric asthmatics [19]. Multimorbidity correlates strongly with mortality, treatment nonadherence, and refractory asthma [14, 15, 16, 17, 18, 19, 20], necessitating Multidimensional Assessment and Management (MDA) strategies [21]. Polypharmacy risks (drug interactions, adverse effects) and age‐related pharmacokinetic alterations demand tailored therapeutic approaches beyond conventional asthma protocols [22].

Hematological analyses revealed that the total number of lymphocytes in the blood circulation begins to decline in youth and reaches a very low level after the age of 60 [23]. Immunosenescence (thymic involution impairing B/T‐cell function) forms a unique immune microenvironment for geriatric asthma. During the aging process, a persistent chronic mild inflammatory state will occur, which is called “inflammatory aging”, resulting in an increase in neutrophils and inflammatory indicators in the airways of geriatric patients compared with younger patients [24]. Inflammation may reduce the adaptability of the immune response, resulting in a corresponding decline in immune system function in geriatric populations, which is called “immune aging” [25]. In this study, we observed significantly higher neutrophil percentages and CRP levels in the geriatric asthma group compared to the nongeriatric asthma group, whereas lymphocyte and eosinophil counts were lower. These findings align with the theories of inflammaging and immunosenescence [26].

In terms of lung function, the results of our study revealed that VC, FVC, FEV_1_, PEF, PEF% predicted, and DLCO% predicted were significantly lowered in the geriatric asthma group compared to the nongeriatric asthma group. Age‐related cumulative structural declines—including diminished lung elastic recoil, reduced chest wall compliance, and impaired small airway patency—contribute to the reduction in FVC, FEV_1_, and FEV_1_/FVC. Lung function reaches its peak at 18–25 years of age and thereafter begins to decline with age [27]. Progressive epithelial apoptosis, fibroblast activation, and inflammatory mediators accumulation drive irreversible airway remodeling, particularly in those with prolonged asthma duration (> 20 years), identified as an independent FAO risk factor (adjusted OR = 8.578, *p* = 0.012) [28].

In this study, a history of asthma > 20 years was found to be an independent risk factor for the presence of FAO in the geriatric asthma cohort, suggesting that the longer the duration of asthma, the more likely they are to develop FAO, which is consistent with the findings of reported studies [11]. In the present study, 45.12% of the geriatric asthmatics who developed FAO had COPD, whereas there were no COPD cases in the reversible airflow obstruction (RAO) group. From this, we can tentatively conclude that geriatric asthmatics with COPD (or what is known as asthma COPD overlap) are more likely to develop FAO. In this study, after excluding ACO cases, a comparative analysis of the geriatric asthma FAO group and the nongeriatric asthma FAO group found that RV/TLC increased significantly in the geriatric asthma FAO group, suggesting that small airway dysfunction and lung hyperinflation are intrinsic markers of aging‐related asthma. This may be due to the progressive loss of elastic retraction force in the lungs and the decoupling of the air cavity and parenchyma with age, which will intensify as the duration of asthma increases [29]. It has been shown that FAO in asthma is mainly associated with airway inflammation, including increased numbers of T lymphocytes (mainly CD4+) and eosinophils. In contrast, FAO associated with COPD is associated with a characteristic inflammation consisting of T lymphocytes (mainly CD8+), macrophages, and neutrophils [30]. FAO in geriatric asthmatics is mainly due to airway remodeling and neutrophil patterns, which are similar to COPD patients [31]. In an Italian epidemiologic study on the general population, asthma and COPD overlapped in up to 61% of asthmatics over 65 years of age [32]. The definition and phenotype of asthma‐COPD overlap (ACOS) are currently controversial internationally, and more research on ACOS is needed in the future to help us provide more appropriate treatment for them.

## 5 Limitation

This study has several limitations. First, as a retrospective single‐center analysis with a relatively small sample size, there exists a potential for selection bias, and the generalizability of the findings may be constrained. The findings from a single tertiary hospital in Guangxi may not be fully generalizable to other populations or healthcare settings. Second, key biomarkers, including sputum inflammatory cell counts, serum IgE, and fractional exhaled nitric oxide (FeNO), were unavailable due to institutional data collection protocols. Most critically, the retrospective design precluded standardized assessment of asthma control using validated tools (Asthma Control Questionnaire [ACQ] or Asthma Control Test [ACT]), thus this study cannot comment on the relationship between the identified characteristics and asthma control or severity. Future multi‐center prospective studies with larger cohorts are warranted to validate these findings and establish tailored management strategies for geriatric asthma in southern China.

## 6 Conclusion

This retrospective study delineates distinctive clinical characteristics of geriatric asthma. Overall, geriatric asthmatics were predominantly female, with cough and expectoration as the main clinical manifestations, and a higher proportion of asthma patients with a course of more than 20 years. Compared with nongeriatric asthma, geriatric asthma patients exhibited elevated neutrophil percentages and CRP levels, coupled with reduced lymphocytes/eosinophils and poorer lung function metrics (VC, FVC, FEV_1_, and PEF). Geriatric asthmatics also showed prolonged hospitalization and heightened sensitization to particulate/irritant allergens. Furthermore, the longer the duration of geriatric asthma, the more likely they were to develop FAO.

## Author Contributions

X.J. proposed the scientific question and designed this study. M.G. and Q.S. analyzed the data, prepared the tables and figures, and wrote the manuscript. X.J., Y.H., and DYT participated in the data analysis and edited the manuscript. All the authors have read and approved the final version of the manuscript.

## Funding

This research was supported by Guangxi Young Talent Reseach Project (grant number: 2080166).

## Ethics Statement

The Ethics Committee of the First Affiliated Hospital of Guangxi Medical University approved the data collection of this retrospective study and granted the exemption from informed consent (Approval Number: 2025‐E0047). All study procedures strictly adhered to the ethical principles of the Declaration of Helsinki. We acquired the right to use the case base of the First Affiliated Hospital of Guangxi Medical University.

## Conflicts of Interest

The authors declare no conflicts of interest.

## Supporting information


**Table S1:** Pulmonary function of geriatric asthma cohort and nongeriatric asthma cohort (based on pooled results from multiple imputation (5 imputations)).
**Table S2:** Pooled analysis of pulmonary function after multiple Imputation.
