# Supplementary material for: Clinical Characteristics of Geriatric Asthma in Guangxi of China: A Retrospective Comparative Descriptive Study
Source: Aging Med (Milton). 2026 Apr 30;9(2):148–57. doi: 10.1002/agm2.70083 (PMC13163941; doi:10.1002/agm2.70083)
Supplement: Supplementary file 1 — Table S1: Pulmonary function of geriatric asthma cohort and nongeriatric asthma cohort (based on pooled results from multiple imputation (5 imputations)). Table S2: Pooled analysis of pulmonary function after multiple Imputation. [file AGM2-9--s001.docx]

**Clinical characteristics of geriatric asthma in Guangxi of China: a retrospective comparative descriptive study**

**Table S1** Pulmonary function of geriatric asthma cohort and non- geriatric asthma cohort(based on pooled results from multiple imputation(5 imputations))

| Variables | Geriatric asthma(n=130) | Non-geriatric asthma(n=130) | *p* value |
| --- | --- | --- | --- |
| VC (L) | 2.08±0.70 | 3.02±0.90 | <.001 |
| FVC (L) | 2.07±0.70 | 3.00±0.94 | <.001 |
| FEV1(L) | 1.24±0.57 | 1.94±0.83 | <.001 |
| FEV1% prediction | 66.49±24.17 | 65.73±24.62 | .81 |
| PEF(L/S) | 3.59±1.73 | 4.98±2.20 | <.001 |
| PEF% prediction | 61.52±24.77 | 67.77±25.95 | .06 |
| Pre-bronchodiator FEV1/FVC(%)  post-bronchodilator FEV1/FVC(%) | 59.96±14.84  62.11±12.94 | 63.48±15.33  66.12±13.89 | .08  .03 |
| DLCO%pred  RV/TLC（%） | 74.29±26.86  49.62±13.67 | 88.37±26.80  36.38±9.96 | <.001  <.001 |

Note：The pooled standard deviations presented in this table were derived from multiple imputation analyses following Rubin's combining rules

Abbreviations: VC: Vital Capacity; FVC: Forced vital capacity; FEV₁,Forced Expiratory Volume in the 1^st^ second; PEF, Peak Expiratory Flow; FVC, Forced vital capacity; DLCO, diffusing capacity of the lung for carbon monoxide; RV, Residual Volume; TLC, Total Lung Capacity..

**Table S2** Pooled analysis of pulmonary function after multiple Imputation

| Variables | group | N (Original/Imputed) | Missing (%) | Original Mean±SD | Imputed Mean±SD | Pooled SD | FMI | RE | Effect size (Cohen's d) [95% CI] |
| --- | --- | --- | --- | --- | --- | --- | --- | --- | --- |
| VC (L) | geriatric asthma | 111/130 | 14.6 | 2.06±0.65 | 2.08±0.70 | 0.70 | 0.172 | 0.967 | -1.226 [-1.51, -0.95] |
|  | non- geriatric asthma | 123/130 | 5.4 | 3.02±0.88 | 3.02±0.90 | 0.90 | 0.019 | 0.996 |  |
| FVC (L) | geriatric asthma | 111/130 | 14.6 | 2.05±0.64 | 2.07±0.70 | 0.70 | 0.084 | 0.984 | -0.805[-1.18, -0.40] |
|  | non- geriatric asthma | 123/130 | 5.4 | 3.00±0.93 | 3.00±0.94 | 0.94 | 0.020 | 0.996 |  |
| FEV1(L) | geriatric asthma | 111/130 | 14.6 | 1.22±0.49 | 1.24±0.57 | 0.57 | 0.159 | 0.969 | -0.675 [-1.07, -0.28] |
|  | non- geriatric asthma | 123/130 | 5.4 | 1.94±0.81 | 1.94±0.83 | 0.83 | 0.057 | 0.989 |  |
| FEV1% prediction | geriatric asthma | 111/130 | 14.6 | 65.81±22.94 | 66.49±24.17 | 24.17 | 0.088 | 0.983 | 0.017 [-0.24, 0.27] |
|  | non- geriatric asthma | 123/130 | 5.4 | 65.40±23.84 | 65.73±24.62 | 24.62 | 0.065 | 0.987 |  |
| PEF(L/S) | geriatric asthma | 111/130 | 14.6 | 3.53±1.59 | 3.59±1.73 | 1.73 | 0.099 | 0.981 | -0.756 [-1.02, -0.49] |
|  | non- geriatric asthma | 123/130 | 5.4 | 4.96±2.12 | 4.98±2.20 | 2.20 | 0.070 | 0.986 |  |
| PEF% prediction | geriatric asthma | 111/130 | 14.6 | 60.71±24..59 | 61.52±24.77 | 24.77 | 0.082 | 0.984 | -0.263 [-0.52, -0.01] |
|  | non- geriatric asthma | 123/130 | 5.4 | 67.38±25.95 | 67.77±25.95 | 25.95 | 0.075 | 0.985 |  |
| Pre-bronchodiator FEV1/FVC(%) | geriatric asthma | 111/130 | 14.6 | 59.70±14.42 | 59.96±14.84 | 14.84 | 1.290 | 0.975 | -0.242[-0.50, 0.02] |
|  | non- geriatric asthma | 123/130 | 5.4 | 63.29±15.20 | 63.48±15.33 | 15.33 | 1.100 | 0.978 |  |
| post-bronchodilator FEV1/FVC(%) | geriatric asthma | 111/130 | 14.6 | 61.85±12.81 | 62.11±12.94 | 12.94 | 0.256 | 0.951 | -0.316[-0.57, 0.06] |
|  | non- geriatric asthma | 123/130 | 5.4 | 66.04±13.83 | 66.12±13.89 | 13.89 | 0.114 | 0.978 |  |
| DLCO%pred | geriatric asthma | 111/130 | 14.6 | 73.79±26.55 | 74.29±26.86 | 26.86 | 0.088 | 0.983 | -0.544[-0.81, -0.28] |
|  | non- geriatric asthma | 123/130 | 5.4 | 88.24±26.56 | 88.37±26.80 | 26.80 | 0.094 | 0.982 |  |
| RV/TLC（%） | geriatric asthma | 111/130 | 14.6 | 49.93±13.28 | 49.62±13.67 | 13.67 | 0.255 | 0.951 | 1.164 [0.89, 1.44] |
|  | non- geriatric asthma | 123/130 | 5.4 | 36.37±9.94 | 36.38±9.96 | 9.96 | 0.031 | 0.994 |  |

Note: VC: Vital Capacity; FVC: Forced vital capacity; FEV₁,Forced Expiratory Volume in the 1^st^ second; PEF, Peak Expiratory Flow; FVC, Forced vital capacity; DLCO, diffusing capacity of the lung for carbon monoxide; RV, Residual Volume; TLC, Total Lung Capacity. FMI: Fraction of missing information; RE: Relative efficiency; Significant difference between original and imputed values (p<0.05, paired t-test); Effect sizes calculated using pooled standard deviations
